# Long-term clinical course and outcomes of immunoglobulin G4-related lung disease

**DOI:** 10.1186/s12931-020-01542-6

**Published:** 2020-10-19

**Authors:** Jieun Kang, Shinhee Park, Eun Jin Chae, Joon Seon Song, Hee Sang Hwang, Sun Jong Kim, Tae Jun Song, Myung-Whan Kim, Jin Woo Song

**Affiliations:** 1 Department of Pulmonary and Critical Care Medicine, Asan Medical Center, University of Ulsan College of Medicine, 88 Olympic-Ro 43-gil, Songpa-gu, Seoul, 05505 Republic of Korea; 2 Division of Pulmonary and Critical Care Medicine, Department of Internal Medicine, Ilsan Paik Hospital, Inje University College of Medicine, Goyang, Republic of Korea; 3 Department of Pulmonary, Allergy and Critical Care Medicine, Gangneung Asan Hospital, Gangneung, Republic of Korea; 4 Department of Radiology, Asan Medical Center, University of Ulsan College of Medicine, Seoul, Republic of Korea; 5 Department of Pathology, Asan Medical Center, University of Ulsan College of Medicine, Seoul, Republic of Korea; 6 Division of Pulmonary and Critical Care Medicine, Konkuk University Hospital, Seoul, Republic of Korea; 7 Department of Gastroenterology, Asan Medical Center, University of Ulsan College of Medicine, Seoul, Republic of Korea

**Keywords:** Immunoglobulin G4-related disease, Treatment outcome, Lung function, Chest computed tomography

## Abstract

**Background:**

Immunoglobulin G4-related lung disease (IgG4-RLD) is the pulmonary manifestation of a systemic fibroinflammatory disease characterized by lymphoplasmacytic infiltration with an abundance of IgG4-positive plasma cells. Long-term clinical course and outcomes of IgG4-RLD remain unclear. We aimed to identify clinical characteristics, treatment outcomes, and longitudinal pulmonary function changes in patients with IgG4-RLD according to the radiologic classification.

**Methods:**

Chest computed tomography findings of 37 subjects were classified into five subtypes: solid nodular, bronchovascular, alveolar interstitial, round ground glass opacity, and alveolar consolidative. Radiologic treatment outcomes and longitudinal pulmonary function changes were compared among the different radiologic subtypes.

**Results:**

The mean age of the subjects was 55.6 years, and 78.4% were male. Among the five radiologic subtypes, alveolar consolidative and solid nodular type were most common, accounting for approximately 29.7% each of the total cases. Prednisone with or without azathioprine was administered to 31 patients (median treatment duration 14 months). In the treated patients, serial images showed complete response or partial response in 77.4%. However, relapse was documented in 25.0% of those who showed complete or partial response. In patients whose longitudinal lung function data were available (n = 20), the lung function was found to be stable during follow-up. Alveolar consolidative type showed the highest complete response rate, whereas alveolar interstitial type showed the lowest response rate, either complete or partial.

**Conclusions:**

Most patients showed a favorable outcome with regards to radiologic improvement and maintenance of pulmonary function; however, the response differed according to the radiologic subtype.

## Background

Immunoglobulin G4-related disease (IgG4-RD) is an increasingly recognized systemic fibroinflammatory disease characterized by lymphoplasmacytic infiltration with an abundance of IgG4-positive plasma cells [1]. It can affect various organs including the pancreas, hepatobiliary system, kidney, retroperitoneum, and lacrimal and salivary glands [2]. Lung involvement can either be the sole manifestation or part of multiple organ involvements [3]. IgG4-related lung disease (IgG4-RLD) has heterogeneous presentations involving airway or interstitium, in addition to tumefactive parenchymal lesions, as reported previously [4–9]. As a result, diagnosis is challenging, and the natural course of the disease is not well defined because most previous literature on this topic is based only on case reports.

Although IgG4-RLD has various manifestations, one previous study found common radiologic features of IgG4-RLD which correlated with pathologic specimens [10]. Inoue et al. classified IgG4-RLD into four subtypes based on a predominant radiologic finding: solid nodular, bronchovascular, alveolar interstitial, and round ground glass opacity (GGO) [10]. In addition to these four subtypes, several case reports suggested alveolar consolidative type as one of the imaging findings of IgG4-RLD [11–13]. However, whether the clinical course of each subgroup is different from one another has not been investigated. Particularly, treatment responses with regards to radiological improvement and long-term pulmonary function changes according to the different radiologic subtypes are yet to be determined. Therefore, this study aimed to identify clinical characteristics, treatment outcomes, and longitudinal pulmonary function changes in patients with IgG4-RLD according to the radiologic classification.

## Methods

### Study design and patients

This retrospective observational study included 37 patients diagnosed with IgG4-RLD at two tertiary care centers in Seoul, South Korea, between March 2004 and August 2016. Lung involvement was confirmed by either lung biopsy (n = 27) or clinical diagnosis (n = 10). Lung biopsy specimens (13 surgical, 10 percutaneous needle, and 4 transbronchial lung biopsy) were independently reviewed by two pathologists (JS Song and HS Hwang) and diagnoses were achieved by consensus. The pathologic diagnosis was in accordance with the 2012 consensus statement on the pathology of IgG4-RD [14]. In brief, the diagnostic criteria included characteristic histopathological features such as dense lymphoplasmacytic infiltrate, storiform fibrosis, and obliterative phlebitis, as well as an increased number of IgG4-positive plasma cells (> 50/high power field) or an elevated IgG4:IgG ratio (> 40%) in the tissue. The clinical diagnostic criteria were as follows: (1) extrapulmonary involvement of IgG4-RD confirmed by biopsy in accordance with the 2012 consensus statement [14], (2) chest computed tomography (CT) findings suggestive of lung involvement of IgG4-RD, and (3) absence of alternative causes for the CT findings. In all cases, the final diagnosis was made via multidisciplinary discussion, after considering pathological, clinical, and radiological findings altogether. This study was conducted in accordance with the amended Declaration of Helsinki and the protocol was approved by the Institutional Review Board of Asan Medical Center (2017-0352). Informed consent was waived due to the retrospective nature of the study.

### Assessment of chest CT images

**Fig. 1:**
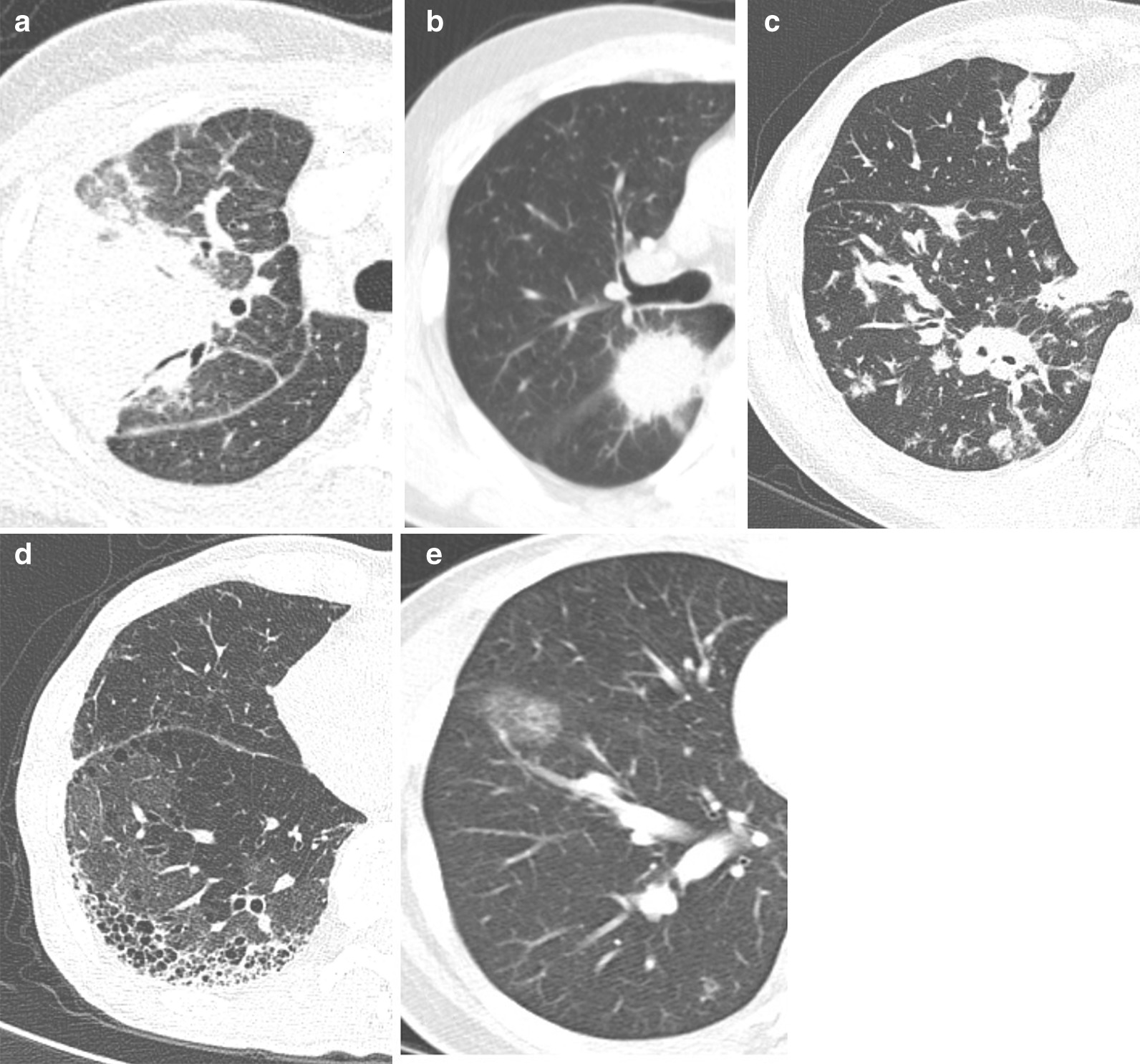
Radiologic subtypes of IgG4-RLD. **a** Alveolar consolidative type, distinguished by airspace filling opacities obscuring vasculature in a segmental or lobar distribution; **b** Solid nodular type, characterized by a lung nodule or mass; **c** Bronchovascular type, characterized by a thickening of bronchovascular bundle and interlobular septa; **d** Alveolar interstitial type, showing reticulation, diffuse GGO, and honeycombing; **e** Round ground glass opacity type, showing multiple round-shaped GGO lesions

Chest CT was performed at the time of diagnosis of lung involvement in all study patients. The CT images were reviewed by a chest radiologist (EJ Chae). The radiologic subtype was determined based on a predominant radiologic feature shown on the CT scan and classified into five subtypes: bronchovascular, solid nodular, round GGO, alveolar interstitial, and alveolar consolidative type, mostly based on the classification suggested by Inoue et al. [10]. Bronchovascular type was characterized by a thickening of bronchovascular bundle and interlobular septa; solid nodular type by a lung nodule or mass; round GGO type by multiple round-shaped GGO lesions; alveolar interstitial type by reticulation, diffuse GGO, and honeycombing; and alveolar consolidative type, distinguished by airspace filling opacities obscuring vasculature in a segmental or lobar distribution. The representative image of each subtype is illustrated in Fig. 1.

### Assessment of clinical courses

To evaluate the clinical course, baseline and follow-up CT images were compared. For patients who received treatment, initial outcome was defined as a radiologic response achieved before relapse or at the end of the treatment. For those who did not receive treatment, it was defined as a radiologic outcome shown before progression or at the end of the follow-up. The treatment outcomes were classified into complete response (CR), partial response (PR), stable disease (SD), and progressive disease (PD). The definition for each treatment outcome, which is partly based on RECIST criteria [15], is given in the Additional file 1.

Relapse was defined as the development of a new lesion or return of abnormal findings after initial CR or PR [16]. In patients who experienced relapse, treatment response for the relapsed disease was evaluated with the same method used for the initial outcome evaluation.

### Pulmonary function test

Forced expiratory volume in one second (FEV_1_), forced vital capacity (FVC), diffusing capacity of the lung for carbon dioxide (DL_CO_), and total lung capacity (TLC) were measured according to the American Thoracic Society (ATS) and European Respiratory Society (ERS) recommendations [17–20]. The measurements were expressed as percentages of the normal predicted values.

To assess the pulmonary function changes, FEV_1_, FVC, TLC, and DL_CO_ values measured at the time of diagnosis and after 1 year were compared in each subgroup. Changes from the baseline values were calculated as follows: (measurement at 1 year − measurement at baseline)/measurement at baseline × 100 (%). For the categorical assessment, FVC changes were classified as follows: an increase of 10% or more from the baseline FVC was defined as improvement; a decrease of 10% or more was defined as deterioration; FVC change that did not meet both the above criteria was defined as no change. In addition, longitudinal pulmonary function changes were evaluated for FEV_1_, FVC, TLC, and DL_CO_ in patients whose pulmonary function were measured at each follow-up visit up to 30 months.

### Statistical analysis

All values were expressed as means and standard deviations (or medians and interquartile ranges) for continuous variables or as percentages for categorical variables. A linear mixed model was used to assess the longitudinal pulmonary function changes during the follow-up. All statistical analyses were performed using SPSS version 21 (IBM Corporation, Armonk, NY, USA).

## Results

### Baseline characteristics

Baseline clinical characteristics of patients who were diagnosed with IgG4-RLD are shown in Table 1. The mean age of the total 37 patients was 55.6 years and 78.4% were male. Elevated serum IgG4 (> 135 mg/dL) level was found in 15 patients (40.5%). Extrapulmonary organ involvement was observed in 16 (43.2%) patients, and the most commonly involved organ was the pancreas (62.5%), followed by the retroperitoneum (31.3%). Among patients with extrapulmonary organ involvement (n = 16), seven (43.8%) presented abnormalities in the lung and extrapulmonary organ simultaneously and eight (50.0%) developed lung involvement during their follow-up of extrapulmonary IgG4-RD. In one patient (6.3%), lung involvement was diagnosed prior to autoimmune pancreatitis which was diagnosed 20 months later. Among the patients who underwent surgical lung biopsy, typical lymphoplasmacytic infiltration, storiform fibrosis, and obliterative phlebitis were observed in 13 (100.0%), 8 (61.5%) and 7 (53.8%) patients, respectively.

**Table 1:** Baseline characteristics of patients with IgG4-RLD

	n = 37
Age, years	55.6 ± 15.0
Male sex	29 (78.4)
Smoking status	
Current smoker	9 (24.3)
Ex-smoker	19 (51.4)
Never smoker	9 (24.3)
Amount of smoking, pack-years	30.7 ± 33.2
Comorbidities	
Hypertension	9 (24.3)
Diabetes	6 (16.2)
Stroke	1 (2.7)
Underlying lung disease	
Tuberculosis sequelae	7 (18.9)
Chronic obstructive pulmonary disease	2 (5.4)
Pneumoconiosis	1 (2.7)
Serum IgG total, mg/dL	1935.7 ± 741.1
Serum IgG4, mg/dL	158.9 ± 129.5
Serum albumin globulin ratio	0.9 ± 0.4
Presence of extrapulmonary organ involvement	16 (45.9)
Pancreas	10 (62.5)
Retroperitoneum	5 (31.3)
Biliary tract	3 (18.8)
Kidney	3 (18.8)
Lacrimal glands	3 (18.8)
Others^a^	8 (43.8)
Pulmonary function test (n = 30)	
Normal	11 (36.7)
Restrictive	10 (33.3)
Obstructive	9 (30.0)
FVC, %pred	82.4 ± 17.5
FEV_1_, %pred	81.4 ± 18.0
FEV_1_/FVC	0.8 ± 0.1
DL_CO_, %pred	75.5 ± 20.7
TLC, %pred	85.2 ± 13.67
Bronchoalveolar lavage (n = 13)^b^	
White blood cell, /µL	447.7 ± 405.6
Neutrophil (%)	8.3 ± 14.2
Lymphocyte (%)	16.1 ± 13.0
Radiologic subtype	
Alveolar consolidative	11 (29.7)
Solid nodular	11 (29.7)
Bronchovascular	8 (21.6)
Alveolar interstitial	5 (13.5)
Round ground glass opacity	2 (5.4)

Data are presented as mean ± standard deviation or number (%)

*IgG4-RLD* immunoglobulin G4-related lung disease, *IgG* immunoglobulin G, *FVC* forced vital capacity, *FEV*_*1*_ forced expiratory volume in 1 s, *DL*_*CO*_ diffusing capacity of the lung for carbon monoxide, *TLC* total lung capacity

^a^Others include adrenal gland (n = 2), salivary gland (n = 1), eyelid (n = 1), mesentery (n = 1), peritoneum (n = 2), colon (n = 1), aorta (n = 1), and prostate (n = 1)

^b^Detailed data regarding differential cell counts are shown in Additional file 1: Table S4

When classified according to the predominant radiologic features, alveolar consolidative and solid nodular type were the most common manifestations, each accounting for 29.7% of the total cases. In contrast, round GGO type was the least common, found only in 5.4% of the patients. Baseline characteristics according to the radiologic classification are shown in Additional file 1: Table S1. Compared to others, alveolar interstitial type tended to include a greater number of patients who were older, male, and ever-smokers, and showed less frequent involvement of extrapulmonary organs. The histopathologic findings according to the radiologic subtypes are described in Additional file 1: Table S2.

### Clinical course

**Fig. 2:**
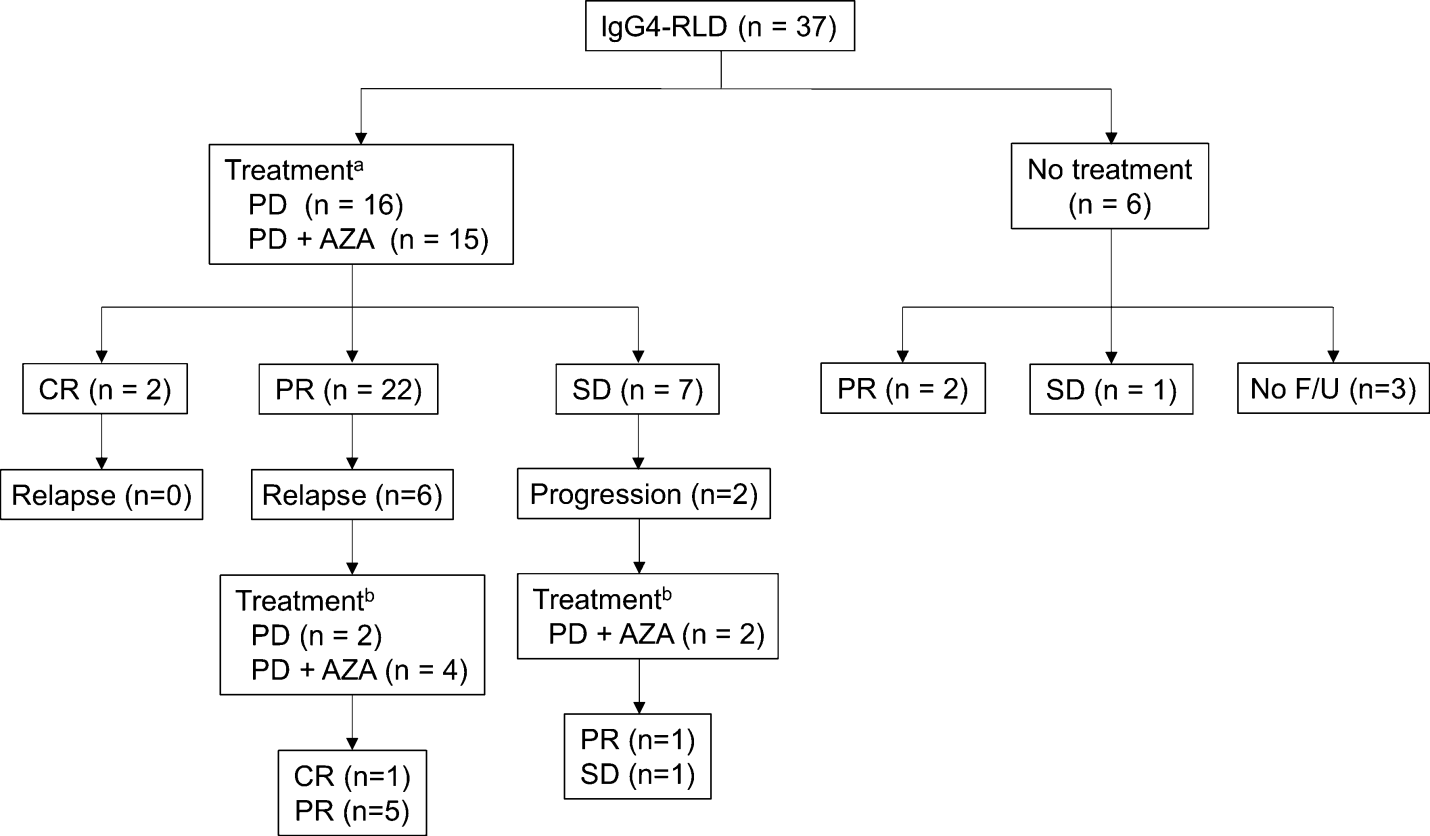
Clinical course of the patients with IgG4-RLD. ^a^Mean initial prednisone dose was 37.8 mg/day (48.5 mg and 27.0 mg for prednisone alone and azathioprine combination groups, respectively). ^b^For patients with relapse, mean initial prednisone dose was 34.1 mg/day (35.0 mg and 33.8 mg for prednisone alone and azathioprine combination groups, respectively). *IgG4*-*RLD* immunoglobulin G4-related lung disease, *PD* prednisone, *AZA* azathioprine, *CR* complete response, *PR* partial response, *SD* stable disease, *F/U* follow up

The median follow-up duration was 38 months in all patients. Figure 2 shows the clinical course of the study patients. In 31 patients, treatment was started with prednisone alone (n = 16) or in combination with azathioprine (n = 15) after diagnosis. Treatment initiation was decided at the discretion of the attending physician based on the patients’ symptoms, imaging findings, and the level of lung function impairment. Whether to treat with a combination of prednisone and azathioprine from the start of treatment was also at the discretion of the treating physician. The median dosage of initial prednisone was 50 mg (interquartile range, 35–60 mg) in patients treated with corticosteroid alone. In patients treated with corticosteroid and azathioprine, the median dosage of prednisone was 30 mg (interquartile range, 20–30 mg). Median treatment duration was 14 months. After treatment initiation, the serial images (median interval 12 months) showed CR in two (6.5%), PR in 22 (71.0%), and SD in seven (22.6%) patients. Among those patients whose initial treatment response was SD, two showed progression at 10 and 11 months after treatment cessation, one achieved PR, and the other showed SD (Fig. 2).

Patients who showed CR after treatment did not show relapse. However, relapse was documented in six patients whose initial treatment response was PR. Among them, three patients were on low-dose steroid maintenance treatment (prednisone 5 mg/day) and the remaining three had ended the initial treatment. Time intervals between relapse and treatment cessation were 4, 29, and 26 months. Patients were treated for relapse with prednisone alone (n = 2) or prednisone with azathioprine (n = 4); patients who had been on steroid maintenance treatment when relapsed were treated with prednisone and azathioprine. CR and PR were achieved in one (16.7%) and five (83.3%) patients, respectively (Fig. 2).

Six patients did not receive treatment; two showed PR without treatment during their 5- and 9-month follow-ups, one showed SD for 36 months, and the remaining three could not be evaluated because of lack of follow-up images.

### Longitudinal pulmonary function changes

Follow-up pulmonary function data at 1 year (± 3 months) were present in 23 patients of which 22 were treated with prednisone with/without azathioprine and one did not receive any treatment. When FVC change at 1 year was assessed in a categorical manner in these 23 patients, improvement, no change, and deterioration were found in 7 (30.4%), 14 (60.9%), and 2 (8.7%) patients, respectively.

**Fig. 3:**
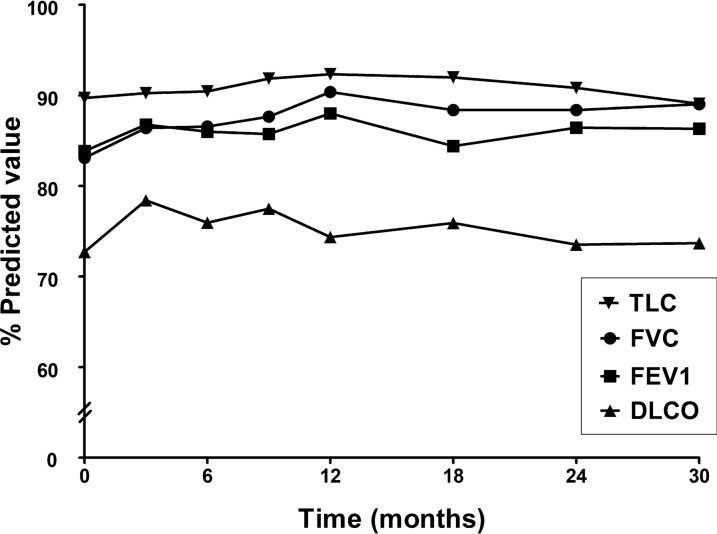
Longitudinal pulmonary function changes in patients who were treated for IgG4-RLD. A linear mixed model was used to calculate the estimated mean changes in lung function parameters. *TLC* total lung capacity, *FVC* forced vital capacity, *FEV*_*1*_ forced expiratory volume at 1 s, *DL*_*CO*_ diffusing capacity of the lung for carbon monoxide

In 20 patients, pulmonary function test data obtained at all follow-up visits up to 30 months were available; all received treatment with prednisone with/without azathioprine. Figure 3 shows the estimated mean longitudinal pulmonary function changes of them with regards to FEV_1_, FVC, DL_CO_, and TLC. As shown in the figure, the pulmonary function parameters stayed stable during the follow-up.

### Treatment responses according to subtypes

Table 2 shows treatment outcomes in patients with IgG4-RLD according to the radiologic subtypes. Compared to others, alveolar consolidative type tended to be treated with steroid alone (Additional file 1: Table S3). When treatment response was evaluated (n = 31), CR was observed only in patients with alveolar consolidative type (20.0%). The proportion of patients who achieved CR or PR was the lowest in the alveolar interstitial type (40.0%) compared with other types (alveolar consolidative, 80.0%; solid nodular, 85.7%; bronchovascular, 87.5%; round GGO, 100.0%). Meanwhile, relapse was more frequent in round GGO (100.0%) and solid nodular types (42.9%), whereas no patient showed relapse in the alveolar interstitial type.

**Table 2:** Comparison of radiologic responses after treatment in patients with IgG4-RDL according to the radiologic classification

	Number of treated patients	Treatment response				Relapse
		Complete response	Partial response	Stable disease	Progressive disease	
Alveolar consolidative	10	2 (20.0)	6 (60.0)	2 (20.0)	0 (0.0)	1 (12.5)
Solid nodular	7	0 (0.0)	6 (85.7)	1 (14.3)	0 (0.0)	3 (50.0)
Bronchovascular	8	0 (0.0)	7 (87.5)	1 (12.5)	0 (0.0)	1 (14.3)
Alveolar interstitial	5	0 (0.0)	2 (40.0)	3 (60.0)	0 (0.0)	0 (0.0)
Round ground glass opacity	1	0 (0.0)	1 (100.0)	0 (0.0)	0 (0.0)	1 (100.0)
Total	31	2 (6.4)	22 (71.0)	7 (22.6)	0 (0.0)	6 (25.0)

Data are presented as numbers (%)

Relapse was defined as a development of a new lesion or return of abnormal findings after initial, complete or partial response

*IgG4-RLD* immunoglobulin G4-related lung disease

Pulmonary function changes for 1 year, in 22 patients, after treatment initiation, are shown according to the radiological classification in Table 3. Alveolar consolidative, bronchovascular, and alveolar interstitial types showed decreased lung functions at the time of diagnosis, but showed greater improvement in FVC, FEV_1_, and DL_CO_ within 1 year compared to the other types. Table 4 shows the categorical changes in FVC for 1 year after treatment (n = 22). Alveolar interstitial type showed the highest improvement rate (100.0%), followed by alveolar consolidative type (60.0%).

**Table 3:** Comparison of baseline values and lung function changes after treatment according to the radiologic classification

	Total	Alveolar consolidative	Solid nodular	Bronchovascular	Alveolar interstitial	Round ground glass opacity
Number of patients	22	5	5	7	4	1
FVC at diagnosis, %pred	80.4 ± 16.3	72.8 ± 25.4	93.6 ± 8.0	90.1 ± 11.7	71.3 ± 3.4	101.0
FEV_1_ at diagnosis, %pred	81.1 ± 17.5	72.0 ± 21.3	91.2 ± 17.4	82.7 ± 14.9	83.8 ± 18.7	90.0
FEV_1_/FVC at diagnosis	76.0 ± 12.3	78.2 ± 20.3	76.4 ± 7.4	72.0 ± 7.2	82.0 ± 14.4	67.0
DL_CO_ at diagnosis, %pred	73.7 ± 22.5	64.8 ± 15.8	91.6 ± 12.8	78.3 ± 15.6	55.3 ± 21.1	93.0
TLC at diagnosis, %pred	85.6 ± 14.0	77.3 ± 14.9	98.7 ± 11.7	82.3 ± 11.0	78.0 ± 3.5	96.0
ΔFVC at 1 year, %	8.9 ± 11.6	19.1 ± 15.5	0.4 ± 1.6	3.9 ± 9.3	15.2 ± 7.1	0.0
ΔFEV_1_ at 1 year, %	5.5 ± .2	10.2 ± 15.5	2.5 ± 3.6	4.2 ± 12.2	7.4 ± 4.4	-2.2
ΔFEV_1_/FVC at 1 year, %	− 3.8 ± 9.6	− 7.9 ± 5.3	− 1.2 ± 6.8	2.8 ± 7.6	− 13.3 ± 13.1	− 4.5
ΔDL_CO_ at 1 year, %	3.9 ± 16.0	8.9 ± 31.1	− 0.4 ± 8.2	11.2 ± 10.8	1.5 ± 9.0	0.0
ΔTLC at 1 year, %	1.8 ± 8.5	11.1 ± 12.5	− 2.9 ± 3.1	− 1.6 ± 2.3	0.9 ± 8.7	3.1

Data are presented as mean and standard deviations

Change in each pulmonary function parameter was calculated as follows: (measurement after 1 year − measurement at baseline)/measurement at baseline × 100 (%)

*IgG4-RLD* immunoglobulin G4-related lung disease, *FVC* forced vital capacity, *FEV*_*1*_ forced expiratory volume in 1 s, *DL*_*CO*_ diffusing capacity of the lung for carbon monoxide, *TLC* total lung capacity

**Table 4:** Comparison of the categorical changes in FVC after treatment according to the radiologic classification

	Number of treated patients	FVC change		
		Improvement	No change	Deterioration
Alveolar consolidative	5	3 (60.0)	2 (40.0)	0 (0.0)
Solid nodular	5	0 (0.0)	5 (100.0)	0 (0.0)
Bronchovascular	7	0 (0.0)	5 (71.4)	2 (28.6)
Alveolar interstitial	4	4 (100.0)	0 (0.0)	0 (0.0)
Round ground glass opacity	1	0 (0.0)	1 (100.0)	0 (0.0)
Total	22	7 (31.8)	13 (59.1)	2 (9.1)

*FVC* forced vital capacity, *IgG4-RLD* immunoglobulin G4-related lung disease

## Discussion

In the present study, we evaluated the characteristics of patients with IgG4-RLD, their long-term clinical course, and treatment outcomes according to the radiologic subtypes. CR or PR was achieved in 77.4% of the treated patients although relapse among them was frequent as 25.0%. Treatment outcomes differed according to the radiologic subtype; alveolar consolidative type showed the highest CR rate whereas alveolar interstitial type showed the lowest number of patients who achieved CR or PR.

IgG4-RD is a systemic inflammatory disease that can involve multiple organs. Lung involvement was first recognized in 2004 [21, 22]. Given the rarity of the condition, it is difficult to ascertain the incidence or prevalence of IgG4-RLD [3]. However, it has been reported that lung involvement is found in as many as 54% of patients with autoimmune pancreatitis [23]. Notably, approximately 50% of the patients were diagnosed with IgG4-RLD without the involvement of any other organ in our study. If lung involvement is investigated in the presence of confirmed IgG4-RD in another organ, the diagnosis of IgG4-RLD may not be so difficult. However, when the lung is the only organ involved, diagnosis becomes much more challenging due to its multifarious radiologic manifestation [3] and the difficulty in detecting a typical characteristic histopathologic feature such as storiform fibrosis in IgG4-RLD [2, 14]. Thus, there is a possibility of underdiagnosis of the disease.

The purpose of IgG4-RLD treatment includes reducing respiratory symptoms and preventing disease progression that may lead to lung fibrosis and subsequent respiratory function decline [24]. Nevertheless, long-term clinical course and treatment outcomes have not been evaluated to date, and differences in these according to the radiologic subtypes remain unclear. According to the results of our study, the prognosis was favorable overall although differences were noted between the subgroups with regards to radiologic improvement and maintenance of lung function. Given the favorable clinical course in IgG4-RLD shown in our study, differentiating from other disorders that mimic IgG4-RLD but have different outcomes should be emphasized. Sarcoidosis involves multiple organs and may have similar image findings such as lymphadenopathy and thickening of peribronchovascular bundles [24, 25]. Lung lesions in multicentric Castleman’s disease and ANCA-associated vasculitis may show IgG4-positive lymphoplasmacytic infiltration [24]. In particular, malignancy should be considered as a differential diagnosis because peritumoral IgG4-positive cell infiltration is often observed in the periphery of a cancer [14].

In our study, five patients (13.5%) were categorized as alveolar interstitial type, of which four demonstrated lung involvement only. Isolated IgG4-related interstitial pneumonia has been reported previously [26, 27]. The rate of patients achieving CR or PR was the lowest in the alveolar interstitial type (40.0%). However, no patients showed PD or relapse. Furthermore, when the lung function changes were assessed in this type (4 out of 5 patients), an improvement in lung function parameters was observed. The FVC was higher than the baseline value by more than 10% in all patients. Thus, alveolar interstitial type does not seem to convey a particularly worse prognosis when compared with the other types.

Baseline pulmonary function test results and their longitudinal changes were different according to the radiologic classification. Alveolar consolidative, bronchovascular, and alveolar interstitial types showed overall lower values at the time of diagnosis compared to the other types. In theory, these types may result in poorer lung function compared to the others due to a larger area involved in alveolar consolidative type, bronchial narrowing in bronchovascular type, and interstitial thickening in alveolar interstitial type. Despite the lower initial measurements at the time of diagnosis, the lung function parameters improved or at least remained stable in most patients after treatment, suggesting a favorable outcome.

In our study, azathioprine was administered in combination with prednisone in 15 patients. Although various steroid-sparing agents such as azathioprine have been used in the treatment of IgG4-RD [28], evidence supporting their use is limited [1]. As in the cases of our study, whether to use an additional immunosuppressive agent is often decided by the treating physician; it was reported that approximately a half of the experts (46%) suggested that some patients require steroid-sparing immunosuppressive agent in addition to glucocorticoids from the start of treatment whereas others did not agree with the combination treatment [29]. It has also not been determined if immunosuppressive agents are required in patients who relapsed. Further studies are warranted to define the role of steroid-sparing immunosuppressive agents in the treatment of IgG4-RLD.

The strength of our study is that it involved the largest number of patients with IgG4-RLD with their long-term clinical outcomes. To the best of our knowledge, this is the only study that presents the long-term follow-up data with a median follow-up duration of 38 months. However, limitations should also be noted. First, this study was a retrospective study performed at two hospitals and this may question the generalizability of our findings. However, the demographic characteristics of patients [24, 30] and relapse rate [30, 31] were found to be similar to those of previous reports. Second, 10 patients with IgG4-RLD were diagnosed clinically without biopsy. Although there is a possibility that the lung lesions might have not been IgG4-RLD manifestations, lung involvement was confirmed only when there was a pathologically confirmed extrapulmonary IgG4-RD as well as compatible chest CT findings without alternative explanation. Third, some pathology specimens were obtained via bronchoscopic or percutaneous needle biopsy instead of surgical lung biopsy. One may argue non-surgical biopsy specimens are inadequate to make a pathological diagnosis of IgG4-RLD. The optimal biopsy method in IgG4-RLD is not determined. According to the consensus statement on the pathology of IgG4-related disease, needle biopsies often provide sufficient proof for the diagnosis of IgG4-RD and exclusion of competing diagnoses [14]. In addition, the method of biopsy may vary among patients according to the location and radiologic manifestation of the target lesion [2]. In fact, patients who underwent transbronchial lung biopsy in our study had alveolar consolidative (n = 3) and bronchovascular type (n = 1) for which the bronchoscopic approach may be appropriate to obtain tissue. Furthermore, other possible causes for the abnormalities in the lungs such as infection were excluded, and the observed steroid responsiveness supports the diagnosis. Fourth, patients with IgG4-RLD may present with other clinical manifestations which do not fit into the classification we used in this study. Previous case reports have shown that IgG4-RLD may have rare presentations such as pleural effusion [32] and pulmonary vascular involvement [33]. Because of the rarity of the disease, the clinical course and long-term outcomes of these subtypes could not be evaluated in this study.

In conclusion, most patients with IgG4-RLD showed favorable outcomes with regards to radiologic improvement and maintenance of pulmonary functions, although there was a difference according to the radiologic subtype. Further studies are warranted to identify an appropriate treatment strategy that maximizes remission and minimizes relapse in different radiologic subtypes.

## Supplementary information

**Additional file 1: Table S1.** Comparison of baseline characteristics of patients with IgG4-RLD according to the radiologic subtypes.** Table S2.** Histopathologic findings according to the radiologic subtype.** Table S3.** Comparison of treatment in patients with IgG4-RLD according to the radiologic subtypes.** Table S4.** Differential cell counts of bronchoalveolar lavage fluid.

## Data Availability

The datasets used and/or analyzed during the current study are available from the corresponding author on reasonable request.

## Abbreviations

ATS: American Thoracic Society
CR: Complete response
DL_CO_: Diffusing capacity of the lung for carbon dioxide
ERS: European Respiratory Society
FEV_1_: Forced expiratory volume in one second
FVC: Forced vital capacity
GGO: Ground glass opacity
IgG4-RD: Immunoglobulin G4-related disease
IgG4-RLD: Immunoglobulin G4-related lung disease
PD: Progressive disease
PR: Partial response
SD: Stable disease
TLC: Total lung capacity
