# Supplementary material for: Long-term clinical course and outcomes of immunoglobulin G4-related lung disease
Source: Respir Res. 2020 Oct 19;21:273. doi: 10.1186/s12931-020-01542-6 (PMC7574178; doi:10.1186/s12931-020-01542-6)
Supplement: Supplementary file 1 — Additional file 1: Table S1. Comparison of baseline characteristics of patients with IgG4-RLD according to the radiologic subtypes. Table S2. Histopathologic findings according to the radiologic subtype. Table S3. Comparison of treatment in patients with IgG4-RLD according to the radiologic subtypes. Table S4. Differential cell counts of bronchoalveolar lavage fluid. [file 12931_2020_1542_MOESM1_ESM.docx]

**Additional File 1**

**Long term clinical course and outcomes of immunoglobulin G4-related lung disease**

Jieun Kang^1,2*^, Shinhee Park^3*^, Eun Jin Chae^4^, Joon Seon Song^5^, Hee Sang Hwang^5^, Sun Jong Kim^6^, Tae Jun Song^7^, Myung-Whan Kim^7^, Jin Woo Song^1^

^1^Department of Pulmonary and Critical Care Medicine, Asan Medical Center, University of Ulsan College of Medicine, Seoul, Republic of Korea

^2^Division of Pulmonary and Critical Care Medicine, Department of Internal Medicine, Ilsan Paik Hospital, Inje University College of medicine, Ilsan, Republic of Korea

^3^Department of Pulmonary, Allergy and Critical Care Medicine, Gangneung Asan Hospital, Gangneung, Republic of Korea

^4^Department of Radiology, Asan Medical Center, University of Ulsan College of Medicine, Seoul, Republic of Korea

^5^Department of Pathology, Asan Medical Center, University of Ulsan College of Medicine, Seoul, Republic of Korea

^6^Division of Pulmonary and Critical Care Medicine, Konkuk University Hospital, Seoul, Republic of Korea

^7^Department of Gastroenterology, Asan Medical Center, University of Ulsan College of Medicine, Seoul, Republic of Korea

**Methods**

**Definition of treatment response**

For the round GGO and solid nodular types, lesions larger than 1 cm were considered as the target lesions. A complete response (CR) was defined as the disappearance of all target lesions; a partial response (PR) was defined as a minimum of 30% decrease in the sum of the diameters of the target lesions relative to the baseline sum. Progressive disease (PD) was defined as a minimum 20% increase in the sum of the diameters of the target lesions relative to the baseline sum; stable disease (SD) was defined as the absence of CR, PR, or PD. For the alveolar interstitial, bronchovascular, and alveolar consolidative types, side-by-side comparisons of baseline and follow-up CT images were performed to determine whether the disease was stable (SD), resolving (PR), or progressing (PD). When the initial abnormalities disappeared completely, it was considered as a CR.

**Table S1. Comparison of baseline characteristics of patients with IgG4-RLD according to the radiologic subtypes**

|  | Alveolar consolidative | Solid nodular | Bronchovascular | Alveolar interstitial | Round ground glass opacity |
| --- | --- | --- | --- | --- | --- |
| Number of patients | 11 | 11 | 8 | 5 | 2 |
| Age, years | 49.0 ± 17.3 | 56.9 ± 7.5 | 54.8 ± 17.5 | 65.8 ± 17.7 | 62.0 ± 7.1 |
| Male sex | 9 (81.8) | 8 (72.7) | 6 (75.0) | 5 (100.0) | 1 (50.0) |
| Smoking status |  |  |  |  |  |
| Current smoker | 5 (45.5) | 2 (18.2) | 1 (12.5) | 1 (20.0) | 0 (0.0) |
| Ex-smoker | 5 (45.5) | 6 (54.) | 4 (50.0) | 3 (60.0) | 1 (50.0) |
| Never smoker | 1 (9.1) | 3 (27.3) | 3 (37.5) | 1 (20.0) | 1 (50.0) |
| Amount of smoking,  pack-years | 39.1 ± 46.4 | 19.4 ± 9.8 | 12.2 ± 10.7 | 48.3 ± 35.2 | 60.0 |
| Comorbidities |  |  |  |  |  |
| Hypertension | 1 (9.1) | 4 (36.4) | 3 (37.5) | 0 (0.0) | 1 (50.0) |
| Diabetes | 1 (9.1) | 2 (18.2) | 3 (37.5) | 0 (0.0) | 0 (0.0) |
| Stroke | 0 (0.0) | 0 (0.0) | 1 (12.5) | 0 (0.0) | 0 (0.0) |
| Underlying lung disease |  |  |  |  |  |
| Tuberculosis sequelae | 5 (45.5) | 1 (9.1) | 1 (12.5) | 0 (0.0) | 0 (0.0) |
| COPD | 0 (0.0) | 0 (0.0) | 0 (0.0) | 2 (40.0) | 0 (0.0) |
| Pneumoconiosis | 1 (9.1) | 0 (0.0) | 0 (0.0) | 0 (0.0) | 0 (0.0) |
| Serum IgG total, mg/dL (n = 36) | 2057.3 ± 776.5 | 1643.3 ± 880.0 | 2227.5 ± 683.1 | 1940.0 ± 630.1 | 3340.0 |
| Serum IgG4, mg/dL (n = 36) | 142.5 ± 140.5 | 145.5 ± 117.2 | 187.1 ± 126.4 | 150.2 ± 102.8 | 13.0 |
| Serum albumin globulin ratio | 0.9 ± 0.4 | 1.1 ± 0.3 | 0.9 ± 0.4 | 0.9 ± 0.4 | 0.6 |
| Presence of extrapulmonary organ involvement | 4 (36.4) | 5 (45.5) | 5 (62.5) | 1 (25.0) | 1 (50.0) |
| Involved extrapulmonary organ |  |  |  |  |  |
| Pancreas | 2 (18.2) | 4 (36.4) | 3 (37.5) | 1 (20.0) | 0 (0.0) |
| Retroperitoneum | 2 (18.2) | 0 (0.0) | 2 (25.0) | 1 (20.0) | 0 (0.0) |
| Biliary tract | 1 (9.1) | 1 (9.1) | 1 (12.5) | 0 (0.0) | 0 (0.0) |
| Kidney | 2 (18.2) | 0 (0.0) | 1 (12.5) | 0 (0.0) | 0 (0.0) |
| Lacrimal glands | 0 (0.0) | 1 (9.1) | 2 (25.0) | 0 (0.0) | 0 (0.0) |
| Others | 3 (27.3)^a^ | 2 (18.2)^b^ | 2 (25.0)^c^ | 0 (0.0) | 1 (50.0)^d^ |
| Pulmonary function test (n = 30) |  |  |  |  |  |
| Normal, n (%) | 2 (40.0) | 6 (60.0) | 2 (25.0) | 0 (0.0) | 1 (50.0) |
| Obstructive, n (%) | 1 (20.0) | 2 (20.0) | 4 (50.0) | 1 (20.0) | 1 (50.0) |
| Restrictive, n (%) | 2 (40.0) | 2 (20.0) | 2 (25.0) | 4 (80.0) | 0 (0.0) |
| FVC, %pred. | 72.8 ± 25.4 | 83.2 ± 19.7 | 88.4 ± 12.0 | 74.4 ± 7.6 | 98.0 ± 4.2 |
| FEV_1_, %pred. | 72.0 ± 21.3 | 80.7 ± 21.1 | 83.1 ± 13.9 | 82.8 ± 16.3 | 98.0 ± 11.3 |
| FEV_1_/FVC | 0.8 ± 0.2 | 0.8 ± 0.8 | 0.7 ± 0.7 | 0.8 ± 0.1 | 0.7 ± 1.0 |
| DL_CO_, %pred. | 64.8 ± 15.8 | 82.2 ± 23.6 | 79.3 ± 14.5 | 63.2 ± 25.5 | 90.0 ± 4.2 |
| TLC, %pred. | 77.3 ± 14.9 | 101.3 ± 10.9 | 81.8 ± 9.1 | 78.0 ± 3.5 | 96.0 |
| Bronchoalveolar lavage (n = 13) |  |  |  |  |  |
| Number of patients | 4 (36.4) | 1 (9.1) | 4 (50.0) | 3 (60.0) | 1 (60.0) |
| White blood cell, /µL | 780.0 ± 539.1 | 260.0 | 167.5 ± 139.4 | 536.7 ± 270.6 | 160.0 |
| Neutrophil (%) | 22.5 ± 19.7 | 0.0 | 0.8 ± 1.0 | 4.7 ± 5.0 | 1.0 |
| Lymphocyte (%) | 20.5 ± 15.8 | 19.0 | 12.3 ± 15.9 | 14.3 ± 13.7 | 16.0 |
| Eosinophil (%) | 0.8 ± 1.0 | 0.0 | 5.3 ± 6.0 | 4.7 ± 6.4 | 2.0 |
| Basophil (%) | 0.8 ± 1.0 | 0.0 | 0.5 ± 1.0 | 0.3 ± 0.6 | 0.0 |
| Alveolar macrophage (%) | 55.5 ± 19.0 | 81.0 | 81.3 ± 21.2 | 76.0 ± 16.0 | 81.0 |

Data are presented as mean ± standard deviation or numbers (%).

^a^three patients showed IgG4-RD involvement in the adrenal gland, mesentery, and peritoneum, respectively.

^b^one patient had prostate involvement; the other had involvement in the peritoneum and adrenal gland.

^c^one patient showed involvement in the colon and aorta; the other showed involvement in the salivary glands.

^d^one patient showed eyelid involvement.

COPD, chronic obstructive pulmonary disease; DL_CO_, diffusing capacity of the lung for carbon monoxide; FEV_1_, forced expiratory volume in 1 second; FVC, forced vital capacity; IgG, immunoglobulin G; IgG4-RLD, immunoglobulin G4-related lung disease; TLC, total lung capacity.

**Table S2. Histopathologic findings according to the radiologic subtype**

| Radiologic subtype | Histopathologic findings |
| --- | --- |
| Alveolar consolidative | Interstitial fibroinflammatory lesion with widening  Diffuse marked lymphoplasmacytic infiltration  Many lymphoid follicles  Organizing pneumonia pattern in some of the involved airspaces |
| Solid nodular | Dense lymphoplasmacytic infiltration with fibrosis  No or few preserved airspaces |
| Bronchovascular | Lymphoplasmacytic infiltration with fibrosis along the bronchovascular bundles  Numerous bronchovascular lymphoid follicles |
| Alveolar interstitial | Interstitial fibroinflammatory widening with preserved airspace and many lymphoid follicles |
| Round ground glass opacity | Inflammatory cell infiltration with alveolar interstitial thickening |

**Table S3. Comparison of treatment in patients with IgG4-RLD according to the radiologic subtypes**

|  | Alveolar consolidative | Solid nodular | Bronchovascular | Alveolar interstitial | Round ground glass opacity |
| --- | --- | --- | --- | --- | --- |
| Number of treated patients | 10 | 7 | 8 | 5 | 1 |
| Types of treatment, n (%) |  |  |  |  |  |
| Prednisone alone | 8 (80.0) | 4 (57.1) | 3 (37.5) | 1 (20.0) | 0 (0.0) |
| Prednisone with azathioprine | 2 (20.0) | 3 (42.9) | 5 (62.5) | 4 (80.0) | 1 (100.0) |
| Initial dose of prednisone, mean, mg/day |  |  |  |  |  |
| Prednisone alone | 53.8 ± 12.7 | 36.0 ± 21.8 | 50.0 ± 17.3 | 40.0 | – |
| Prednisone with azathioprine | 32.5 ± 3.5 | 25.0 ± 8.7 | 22.0 ± 5.7 | 31.3 ± 4.8 | 30.0 |

Data are presented as numbers (%) or mean ± standard deviation.

**Table S4. Differential cell counts of bronchoalveolar lavage fluid**

| Bronchoalveolar lavage fluid (n = 13) |  |
| --- | --- |
| White blood cell, /µL | 447.7 ± 405.6 |
| Neutrophil (%) | 8.3 ± 14.2 |
| Lymphocyte (%) | 16.1 ± 13.0 |
| Eosinophil (%) | 3.1 ± 4.6 |
| Basophil (%) | 0.5 ± 0.8 |
| Alveolar macrophage (%) | 72.1 ± 19.6 |
